# An Alternative Metabolic Pathway of Glucose Oxidation Induced by Mitochondrial Complex I Inhibition: Serinogenesis and Folate Cycling

**DOI:** 10.3390/ijms262311349

**Published:** 2025-11-24

**Authors:** Roman Abrosimov, Ankush Borlepawar, Parvana Hajieva, Bernd Moosmann

**Affiliations:** 1 Evolutionary Biochemistry and Redox Medicine, Institute for Pathobiochemistry, University Medical Center of the Johannes Gutenberg University, 55128 Mainz, Germany; rabrosim@students.uni-mainz.de; 2 Cellular Adaptation and Bioenergetics, Institute for Translational Medicine, MSH Medical School, 20457 Hamburg, Germany; ankush.borlepawar@medicalschool-hamburg.de (A.B.); parvana.hajieva@medicalschool-hamburg.de (P.H.); 3 Institute for Quantitative and Computational Biosciences, Johannes Gutenberg University, 55128 Mainz, Germany

**Keywords:** fatty acid cycling, futile cycle, glycolytic inhibition, metabolic reprogramming, metformin, mitochondrial disease, NADPH-FADH_2_ axis, oxidative stress, Parkinson’s disease

## Abstract

Inhibition of respiratory chain complex I (NADH dehydrogenase) is a widely encountered biochemical consequence of drug intoxication and a primary consequence of mtDNA mutations and other mitochondrial defects. In an organ-selective form, it is also deployed as antidiabetic pharmacological treatment. Complex I inhibition evokes a pronounced metabolic reprogramming of uncertain purposefulness, as in several cases, anabolism appears to be fostered in a state of bioenergetic shortage. A hallmark of complex I inhibition is the enhanced biosynthesis of serine, usually accompanied by an induction of folate-converting enzymes. Here, we have revisited the differential transcriptional induction of these metabolic pathways in three published models of selective complex I inhibition: MPP-treated neuronal cells, methionine-restricted rats, and patient fibroblasts harboring an NDUFS2 mutation. We find that in a coupled fashion, serinogenesis and circular folate cycling provide an unrecognized alternative pathway of complete glucose oxidation that is mostly dependent on NADP instead of the canonic NAD cofactor (NADP:NAD ≈ 2:1) and thus evades the shortage of oxidized NAD produced by complex I inhibition. In contrast, serine utilization for anabolic purposes and C_1_-folate provision for S-adenosyl-methionine production and transsulfuration cannot explain the observed transcriptional patterns, while C_1_-folate provision for purine biosynthesis did occur in some models, albeit not universally. We conclude that catabolic glucose oxidation to CO_2_, linked with NADPH production for indirect downstream respiration through fatty acid cycling, is the general purpose of the remarkably strong induction of serinogenesis after complex I inhibition.

## 1. Introduction

The mitochondrial respiratory chain is the target of numerous natural and synthetic toxins [1], many of which specifically interfere with complex I (NADH dehydrogenase) function [2,3]. Depending on the precise drug mechanism, blocking complex I may elicit an accumulation of reduced NADH, causing a secondary slowdown of the citric acid cycle [4], potentially resulting in bioenergetic failure, and, secondly, an increased production of reactive oxygen species (ROS), first as signaling molecules (at low concentrations) [5,6], and later as oxidative toxins (at high concentrations) [5,7,8]. Idiopathic and toxin-induced deficiency of complex I are robustly linked to Parkinson’s disease [8,9,10] and potentially other neurodegenerative disorders [11,12]. Concomitantly, complex I is an important therapeutic drug target, with different anti-diabetic substances acting primarily [13,14] or secondarily [15,16] at this large membrane protein complex. However, their action is generally only tolerated when it occurs in metabolically flexible tissues such as the liver and the gut, but spares the brain or the heart, as it seems to be the case for metformin [17].

Understanding the adaptive responses of the liver and other tissues after complex I inhibition is not only relevant for understanding the mechanism of these important drugs and toxins, but is also relevant for the pathobiochemistry of mitochondrial diseases. These diseases comprise a wide spectrum of mostly inherited defects, which often affect the mitochondrial DNA on which all mitochondrial tRNAs and 13 respiratory chain complex subunits are encoded [18]. Since 7 of these 13 subunits, and thus ~2000 of the ~3700 mitochondrially encoded amino acids, belong to complex I [19], it is plausibly explained why generalized mitochondrial translation defects due to tRNA mutations can resemble authentic complex I defects [18,20]. A similar argument applies to findings in experimental mouse models of impaired mitochondrial DNA maintenance, replication, transcription, transcript maturation, and translation, which surprisingly resemble each other and involve complex I insufficiency as a landmark phenotype [21].

Various transcriptomic and metabolomic studies have investigated the metabolic adaptations in the wake of specific or pleiotropic complex I inhibition in different models, including metformin and imeglimin treatment in vitro [22], MPP treatment in vitro [6], and dietary methionine restriction (MetR) in vivo [23]. Despite substantial differences between these models, a series of highly striking, recurrent metabolic changes were observed [24] that were not self-explanatory. In particular, recurrent observations were (i) the induction of the pentose phosphate pathway (PPP) producing NADPH, (ii) the induction of fatty acid biosynthesis with unaltered or likewise induced β-oxidation (“fatty acid cycling”) (iii), a pronounced induction of serine biosynthesis (“serinogenesis”), and (iv) the induction of various folate-metabolizing enzymes.

Recently, we proposed a model to explain the induction of NADPH production (i) and fatty acid cycling (ii) as a purposeful strategy to maintain respiratory chain fueling with electrons despite complex I inhibition, as the coupling of these processes would deliver 12 FADH_2_ per glucose, for feeding the respiratory chain via the electron-transferring flavoprotein (ETF). This type of coupling was called the “NADPH-FADH_2_ axis” and enables an entirely NAD-independent and citric acid cycle-independent oxidation of glucose to CO_2_ [24]. In vivo, this strategy implies a substantial energetic cost to organs generally unaffected by toxin-induced complex I inhibition (e.g., adipose tissue), which would have to carry the bioenergetic load of maintaining respiration in complex I-inhibited tissue (e.g., liver). In consequence, potentially substantial metabolic inefficiency should occur in vivo, which matches the actual observations made after metformin treatment [25], methionine restriction [26,27], and in patients with mitochondrial diseases [28].

As regards the above observations (iii) and (iv), increased serinogenesis has repeatedly emerged as one of the most pronounced, quantitative alterations in response to various types of mitochondrial impairment. In particular, significant induction of serine biosynthetic genes has been shown not only in relation to MPP treatment [29], but also in genetic complex I defects [30], genetic defects in mitochondrial DNA maintenance [31,32,33], mitochondrial transcription [21], mitochondrial translation [21], and mitochondrial protein import [34]. Accordingly increased protein expression and elevated levels of free serine have also been reported in some studies [21,33,35], but a consensus functional interpretation of the serine boost after mitochondrial impairment has not been achieved [21,36,37]. Interestingly, a frequent companion of serinogenesis was the soaring induction of the mitochondrial enzyme methylenetetrahydrofolate dehydrogenase (MTHFD2) [21,23], opening the possibility that serinogenesis might serve the recharging of C_1_-metabolism. In consequence, elevated serinogenesis might serve either catabolic purposes (withdrawing 3-phosphoglycerate) as well as numerous anabolic purposes (ranging from serine-dependent protein synthesis, sphingolipid synthesis, and cysteine/glutathione provision to all pathways dependent on C_1_-metabolism such as purine synthesis). The relative importance of these downstream pathways has remained unsettled, potentially due to the diversity of methods by which mitochondrial impairment had been induced.

To better understand the purpose of serinogenesis (iii) and folate dehydrogenation (iv) after mitochondrial impairment, we have compared these hallmark responses in three largely specific and well-characterized models of complex I inhibition, namely pharmacological MPP treatment in vitro (model 1) [6,29], methionine restriction (MetR) in vivo (model 2) [23,27,38,39,40], and patient fibroblasts in vitro that harbor an NDUFS2 mutation (model 3) [41,42]. Should a coherent picture emerge from these otherwise divergent models, this consensus picture may potentially be generalizable. We found that, in a coupled form, serinogenesis and folate dehydrogenation most likely provide a means of continued glucose oxidation and maintained respiratory chain fueling despite complex I deficiency, which we refer to as the “serine–folate shunt” in the following. Hence, the four major and recurrently arising metabolic alterations (i)–(iv) induced by complex I inhibition all seem to serve the same adaptive purpose of bypassing the triggering complex I blockade.

## 2. Results

### 2.1. The Serine–Folate Shunt

**Figure 1:**
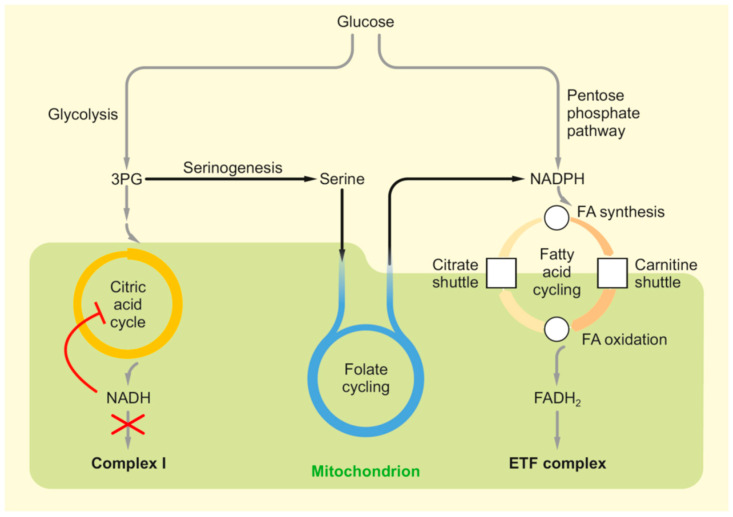
Overview of the serine–folate shunt. The proposed metabolic coupling connects default glycolysis, an 80% NAD-dependent oxidation pathway (left), with the inducible NADPH-FADH_2_ axis (right), which is an emergency metabolic pathway to oxidize glucose independently of oxidized NAD when this cofactor is limiting, as during complex I inhibition (red cross). The serine–folate shunt starts with glycolytic 3-phosphoglycerate (3PG), from which serine is synthesized as key intermediate. The serine carbon skeleton is then completely oxidized to CO_2_ through mostly mitochondrial folate cycling, a process producing predominantly (up to 80%) NADPH. The NADPH is then fueled into the respiratory chain by fatty acid (FA) cycling or serves other purposes. Further details are given in the text.

Inhibition of electron transfer through complex I is typically associated with an increased NADH/NAD ratio [6,41,43], resulting in a slowdown of NAD-dependent oxidative processes such as the citric acid cycle [4] (Figure 1), increased lactate production in the cytosol [20], and other consequences [44]. A frequently made observation in response to rising NADH/NAD ratios is glucose metabolic diversion towards the pentose phosphate pathway (PPP) [45,46], which enables an NAD-independent, but rather NADP-dependent catabolic removal of glucose that is especially relevant after high glucose uptake. Under these conditions, the NADPH produced by the PPP is used for fatty acid biosynthesis but may also be utilized to repair oxidative damage [45,47], as often triggered by complex I inhibition. However, a significant induction of the PPP has also been observed in models of complex I inhibition that typically lack signs of oxidative stress, such as methionine-restricted rats or metformin-treated cells [24].

Based on the observation that not only NADPH-dependent fatty acid biosynthesis was induced in these models, but also oxidative fatty acid degradation (β-oxidation), a coupling of the PPP with the ensuing fatty acid cycling was proposed, as it was determined that the only net redox effect of fatty acid cycling is the conversion of NADPH into respirable FADH_2_ [24]. The resulting metabolic coupling was termed the “NADPH-FADH_2_ axis”. Considering the prominently induced reactions of serine biosynthesis and folate conversion after complex I inhibition in more detail, we have found and describe in the following that a metabolic crosslink can be defined that would also enable a complete oxidation of glucose to CO_2_ by connecting glycolysis (from the branching point 3-phosphoglycerate) with the NADPH-FADH_2_ axis (more specifically, with NADPH) (Figure 1). This bypass, the serine–folate shunt, may sustain glucose degradation even when both complex I and the PPP are functionally impaired (Figure 1).

### 2.2. Induction of the Serine–Folate Shunt in Three Models of Complex I Deficiency

The transcriptional induction of the serine–folate shunt was investigated in data from MPP-treated, differentiated neuronal stem cells (LUHMES) [6], methionine-restricted (MetR) Fischer rat liver [23], and human skin fibroblasts harboring an NDUFS2 mutation [42]. Of note, the MPP-treated cells showed a wider induction of formally significant gene regulatory events than the two other models (compared in Section 4.4), which may relate to the partially maintained stem cell character of these cells, or to a potentially higher intrinsic potency of the applied pharmacological intervention, 10 µM MPP. Hence, *p* values should only be compared within each model, and FC values should be interpreted cautiously.

**Figure 2:**
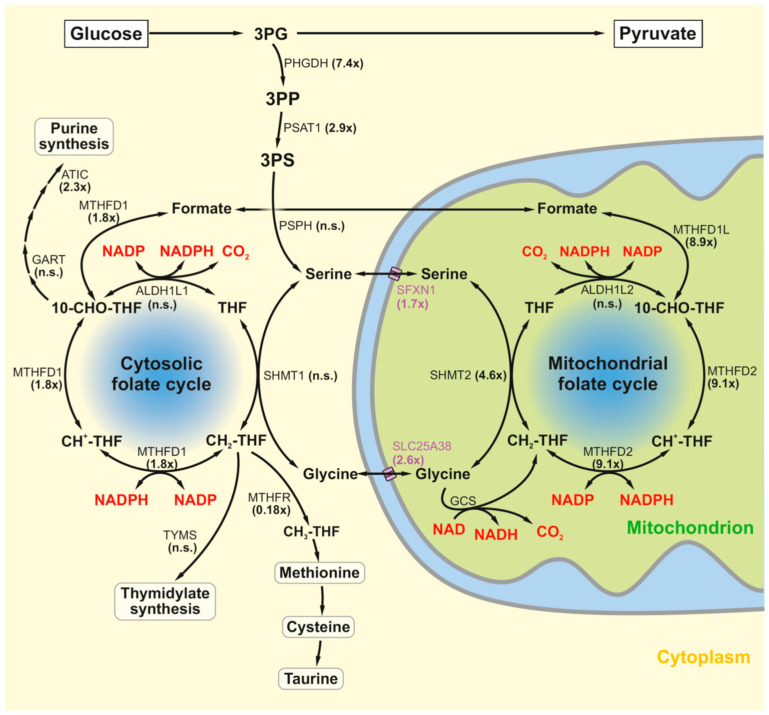
Induction of the serine–folate shunt in human neuronal cells after complex I inhibition with MPP. The reactions of serinogenesis, the cytosolic folate cycle, and the mitochondrial folate cycle are depicted. The transcriptional induction (x-fold) of each enzyme after pharmacological complex I inhibition is indicated in brackets behind each enzyme acronym. A selection of potentially competing reactions is also shown. Sites of NADPH production and CO_2_ formation are highlighted in red. A more complete list of transcriptional changes in all three investigated models of complex I catalytic insufficiency is provided in Table 1. The abbreviations denote the following: 3PG, 3-phosphoglycerate; 3PP, 3-phosphohydroxypyruvate; 3PS, 3-phosphoserine; THF, tetrahydrofolate; CH_2_-THF, methylenetetrahydrofolate; CH^+^-THF, methenyltetrahydrofolate; 10-CHO-THF, 10-formyltetrahydrofolate; n.s., not significant. Enzyme acronyms are decoded in the legend of Table 1.

Serinogenesis was strongly induced in all three models (Table 1), supported by correspondingly altered amino acid levels in identical MPP-treated cells [29] and MetR animals [23]. To track the likely fate of the produced serine, key results from MPP-treated cells are visualized in Figure 2. Following the course of the most visibly induced enzymes, serine would be targeted to enter the mitochondria via SFXN1 and would load mitochondrial tetrahydrofolate (THF) to yield methylene-THF via serine hydroxymethyltransferase 2 (SHMT2). Subsequently, representing the most heavily induced enzyme of the pathway in neuronal cells, methylene-THF would be oxidized to 10-formyl-THF by MTHFD2, a signature enzyme also frequently found among the most highly induced enzymes after mitochondrial impairment in vivo [21,23,31]. MTHFD2 has evolved to also accept NAD as an oxidizing cofactor beyond NADP [48], but complex I inhibition (restricting NAD availability) may render this enzyme mostly dependent on NADP. The produced 10-formyl-THF may then be terminally oxidized to CO_2_ (by ALDH1L2) in the mitochondrion or, preferably, will release formate (through MTHFD1L) for export from the mitochondrion, followed by re-fixation as 10-formyl-THF (by MTHFD1) in the cytosol (to replenish the cytosolic folate cycle), and subsequent cytosolic oxidation by ALDH1L1. To complete the oxidative degradation of serine, the produced, evidently elevated [29] amino acid glycine from the SHMT2 reaction needs to be taken care of. The tentatively assigned, canonic glycine cleavage system was found to be robustly induced in vivo, but unchanged in the fibroblast system. In neuronal cells, its expression was too low to draw reliable conclusions (Table 1).

The cytosolic folate cycle is the source of several substantial outlets that consume C_1_-units, including the synthesis of purines, thymidylate, and methionine, an essential precursor of S-adenosylmethionine, cysteine, and taurine. Still, the enzymes of the cytosolic folate cycle were generally less induced than the mitochondrial enzymes. These patterns in MPP-treated cells were largely recapitulated by the other models (Table 1), but with the difference that the NDUFS2 fibroblasts directed their NADPH production preferably to the mitochondrion, through a significant induction of ALDH1L2. Depending on the purpose of the produced NADPH, its export from the mitochondrion may be crucial, especially if it should be targeted for fatty acid cycling, which starts in the cytosol. Formate export of the above kind would be equivalent to the export of a molecule of NADPH from the mitochondrion, as NADPH production is thereby diverted from the mitochondrion (ALDH1L2) to the cytosol (ALDH1L1). The likely fate of the other mitochondrially produced NADPH is either direct mitochondrial consumption, or indirect export to the cytosol via the IDH1-IDH2 circuit [49]. This circuit seems to be operable preferably under reducing conditions such as hypoxia [50] and glutamine loading [49], which resemble complex I inhibition in producing a high NADH/NAD ratio. Under such conditions, the mitochondrial NADP-dependent enzyme IDH2 works in reverse, generating isocitrate from α-ketoglutarate in an NADPH-dependent fashion [50]. The generated isocitrate is then exported to the cytosol, where cytosolic IDH1 reverses the reaction and generates α-ketoglutarate and NADPH. In MPP-treated cells, IDH1 and IDH2 were significantly induced (Table 1).

### 2.3. Quantitative Metabolic Outcome of the Serine–Folate Shunt

**Scheme 1:**
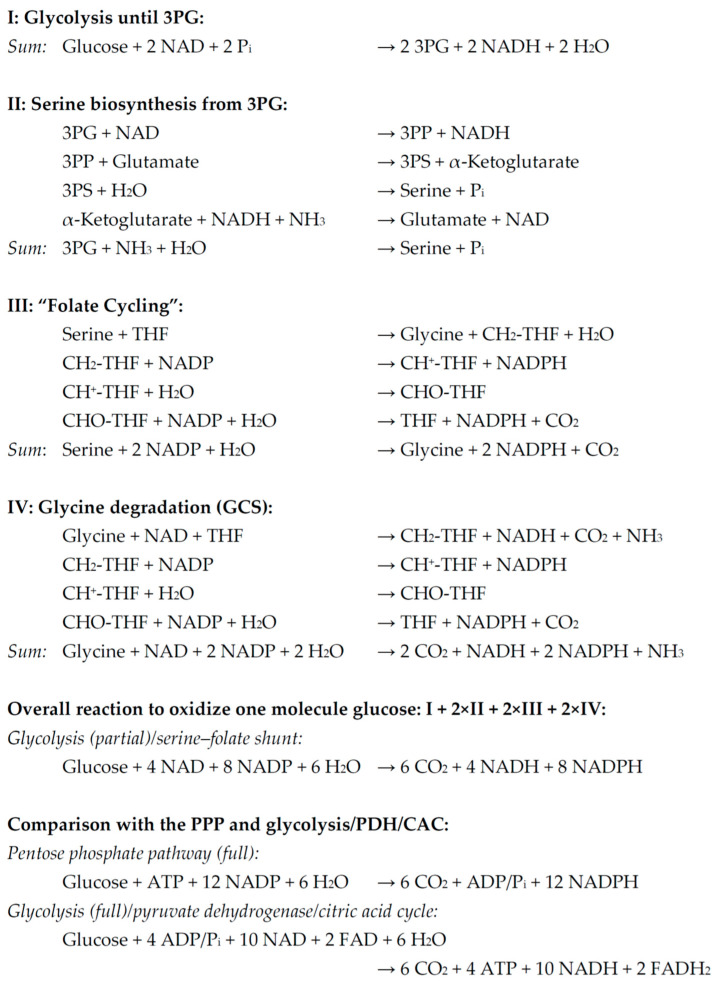
Reaction summary of glucose degradation via 3-phosphoglycerate and the serine–folate shunt. For clarity, charges and released protons (H^+^) were generally omitted.

The metabolite balance of the serine–folate shunt was determined and is depicted in Scheme 1. By itself, the serine–folate shunt is effectively a complete oxidative degradation of 3-phosphoglycerate that runs independently of the citric acid cycle. For reasons of comparability with competing pathways that also provide a complete oxidation of glucose to CO_2_, the following calculations were all normalized on glucose. Hence, the serine–folate shunt was preceded by a partial glycolysis (i.e., the first 7 of 10 reactions) that produce two molecules of 3-phosphoglycerate (Scheme 1, I). To summarize the reactions of serinogenesis (Scheme 1, II), it was assumed that the recycling of α-ketoglutarate to glutamate would be executed by glutamate dehydrogenase in an NADH-dependent mode. “Folate cycling” was defined as the four-reaction set to degrade serine to glycine and CO_2_ (Scheme 1, III). The concluding glycine degradation was supposed to occur via the canonic glycine cleavage system (GCS), even if other metabolic fates of glycine exist (Scheme 1, IV). In toto, the coupling of a partial glycolysis with the serine–folate shunt results in a complete oxidation of glucose to six CO_2_ yielding four NADH and eight NADPH, without any net production or consumption of ATP. For comparison, the complete oxidation of glucose to six CO_2_ via the pentose phosphate pathway (PPP) yields 12 NADPH at a cost of (−1) ATP. Canonic glucose catabolism via glycolysis, pyruvate dehydrogenase (PDH), and the citric acid cycle (CAC) yields 10 NADH, 2 FADH_2_, and 4 ATP (Scheme 1).

**Scheme 2:**
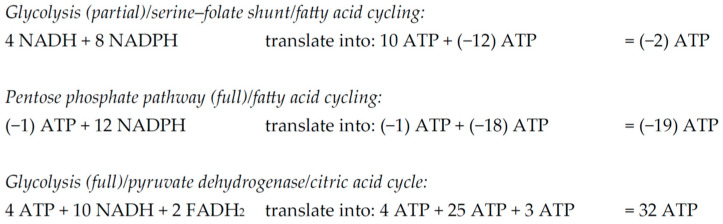
Cofactor balance sheet for three pathways of complete glucose oxidation to CO_2_.

For the determination of the ATP yield to be expected from the oxidation of these reduced cofactors, the following assumptions were made (Scheme 2): operable NADH oxidation (without complex I inhibition) may provide 2.5 ATP [51], any FADH_2_ oxidation may provide 1.5 ATP [51], while NADPH oxidation would proceed via the energetically inefficient process of fatty acid cycling and may thus cost (−1.5) ATP [24]. In consequence, the partially glycolytic serine–folate shunt unveils as an intermediary between the two other pathways. The canonic, complex I-operable pathway is highly efficient and delivers approximately 32 ATP per glucose as expected, whereas the NADPH-FADH_2_ axis yields a negative balance of (−19) ATP as reported before [24]. The partially glycolytic serine–folate shunt is essentially neutral in bioenergetic terms, resulting in a small energetic cost of (−2) ATP (Scheme 2).

### 2.4. Alternative Explanations to Account for Enhanced Serinogenesis and Folate Metabolism After Complex I Inhibition

The key metabolites of the serine–folate shunt (serine, glycine, various folates, and NADPH) may also serve other terminal purposes than the catabolic oxidation of glucose via serine. Hence, a survey of the most notorious candidate reactions was assembled to compare the transcription of the involved genes with the genes of the serine–folate shunt (Table 2). Enzymes to consume serine for anabolic purposes such as serine racemase (SRR), serine palmitoyltransferase (SPTLC) (producing sphingolipids), and cystathionine β-synthase (CBS) (producing cysteine) were unaltered throughout. Transcription of 10-formyl-THF-dependent MTFMT (required for mitochondrial protein synthesis) was also unaltered or reduced, whereas the two consumers of the same cofactor found in purine biosynthesis were mostly induced, and significantly so in MPP-treated cells and MetR rats. To differentiate between increased DNA versus RNA versus purine synthesis, the committed regulatory step of purine synthesis was analyzed (PPAT) together with the purine salvage enzymes APRT and HPRT1. By trend or significantly, these enzymes were induced in MPP-treated cells and MetR rats, but not in NDUFS2-mutant patient fibroblasts. Pyrimidine synthesis, however, did not follow these trends: either one enzyme of thymidylate synthase (TYMS) or the TYMS cofactor recycling enzyme, dihydrofolate reductase (DHFR), were significantly downregulated in all models (Table 2). The committed step of de novo pyrimidine synthesis (CAD) was unaltered except in MetR rats, while methylene-THF-dependent ribonucleotide reductase (RRM) expression behaved variably. These data suggest a selective induction of purine synthesis and perhaps RNA production in MPP-treated cells and MetR rats, but do not appear to mirror an increased demand for DNA synthesis. In the third model, both essential enzymes of DNA building block synthesis were in fact significantly reduced (TYMS and the decisive regulatory subunit RRM2).

The methionine cycle, producing S-adenosylmethionine (SAM) for numerous methylation reactions, is a major efflux path for C_1_-units from the cytosolic folate cycle [52]. Downstream of the methionine cycle are located the biosynthesis of cysteine, glutathione and taurine. In MPP-treated cells, the key enzyme to divert C_1_-units from the pool, methylenetetrahydrofolate reductase (MTHFR), was strongly suppressed, as was the major catalytic methionine adenosyltransferase (MAT2A) for SAM synthesis. Consistently, reduced DNA methylation has been noted in this model [6,53], implying functional relevance. The transcripts of the methionine cycle were mostly unaltered in the other models, but a substantial, 5-fold lower concentration of the metabolite methyl-THF has been noted in MetR rats [23].

NADPH formation is essential for the operability of antioxidant enzymes such as glutathione reductase (GSR), thioredoxin reductase (TXNRD), or methionine sulfoxide reductase (MSR), and oxidative stress frequently resulting from complex I inhibition could be causative to the metabolic induction of NADPH formation [36,47]. Inspection of the transcriptional profile of these NADPH-dependent reductases and of various related, direct antioxidant enzymes including the inducible glutathione-dependent peroxidases (GPXs) and the autonomous redox dismutases, superoxide dismutase (SOD) and catalase (CAT), indicated that severe oxidative stress was elicited in LUHMES cells by MPP, as the according transcripts were broadly and significantly induced. No substantial differences could be detected between primarily mitochondrial enzymes (GPX4, SOD2, TXNRD2, and TXN2) and primarily cytosolic enzymes (GSR, GPX1, SOD1, CAT, TXNRD1, and TXN). In contrast, complex I-deficient patient fibroblasts did not exhibit any broad induction of antioxidant enzymes, while the key cytosolic peroxidase GPX1 was rather reduced. In the MetR in vivo model, two NADPH consumers (GSR and TXNRD1) that canonically recycle other antioxidants [54], were significantly increased, whereas two very important direct antioxidants of the liver (GPX1 and CAT) were significantly reduced (Table 2). These data suggest that oxidative stress is not a universally relevant trigger of NADPH formation via the serine–folate shunt, since the latter was similarly induced in oxidatively stressed (MPP-treated) and unstressed paradigms (MetR rats and patient fibroblasts). The inferred steady-state levels of oxidative stress in MPP-treated LUHMES cells [6,29] and MetR rats [38,39] have been described and confirmed before.

## 3. Discussion

Induction of serinogenesis is a perseverative quantitative phenomenon in response to an impairment of mitochondrial respiration. For instance, enhanced serinogenesis has been described in mice carrying a dominant TWINKLE defect, which causes multiple mtDNA deletions [31,32]; in four additional mouse models of impaired mitochondrial DNA maintenance, transcription and translation (i.e., mice defective in TFAM, POLRMT, LRPPRC, or MTERF4) [21]; and in NDUFS1-knockout mice, an animal model of complex I dysfunction mimicking Leigh syndrome [30]. Serinogenesis has also been found highly induced in methionine-restricted (MetR) mice [55] and rats [23], which exhibit an antioxidative, antidiabetic longevity phenotype involving mitochondrial impairment and metabolic insufficiency [24,27,38,39,55,56], and in long-lived *C. elegans* nematodes with a targeted mitochondrial protein import system defect [34]. Finally, serinogenesis has been described as emerging in numerous cancer cell lines [57], particularly under hypoxic conditions [36].

The transcriptional induction of serinogenesis has in various cases been confirmed to result in increased protein expression [21,35], increased C_1_-folate species [32], and in sometimes substantial quantitative increases in free serine [21,23,29,33,35]. In HEK293 cells with a conditional mtDNA depletion, serine was the most highly induced (~8-fold) of 191 metabolites, accompanied by the upregulation of serine biosynthesis and serine uptake [33]. In MetR rats, free serine levels were strongly increased in all examined tissues (liver: 5.0-fold; quadriceps muscle: 4.2-fold; adipose tissue: 2.6-fold), including in serum (2.7-fold) [23]. In these animals, serine was more substantially altered than the model-generating, restricted amino acid, methionine, and its metabolic descendant, cysteine [23]. Similar effects were observed in a related, but more controlled model of titrated sulfur amino acid restriction [35]. The quantitative nature of the triggered serinogenesis resulting from various types of mitochondrial impairment points to a quantitative metabolic purpose of this response rather than to a merely regulatory phenomenon.

Among the most widely cited explanations for the induction of serine biogenesis and the frequently associated induction of folate metabolism are the general amelioration of oxidative stress through NADPH provision [36], the specific amelioration of oxidative stress through enhanced glutathione synthesis (which is enabled by methylene-THF via methyl-THF, methionine, and cysteine) [31,55], or the provision of 10-formyl-THF for nucleotide biosynthesis and mitochondrial translation [32]. Non-adaptive interpretations describing potentially adverse consequences such as the production of additional NADH have also been proposed [58]. In most cases beyond cancer studies, though, serinogenesis was found to represent an adaptive, beneficial response [31,32,34,36,55]. In the end, it is likely that the purposefulness of serinogenesis and especially the subsequent folate hand-ling may also be influenced by the cell type and the cellular proliferation state [37].

In contrast to the above interpretations that may be summarized as serine-dependent anabolic concepts of building block provision and antioxidant repair, we here propose that serinogenesis effectively mediates an overall catabolic pathway relevant for energy generation (Figure 1). In this concept, serine is the central part of a somewhat meandering glucose degradation pathway that starts with the first seven steps of canonic glycolysis, continues with the three reactions of serinogenesis, and ends with the overall four reactions of the mitochondrial folate cycle plus the reaction sequence of the glycine cleavage system (here counted as one reaction) (Figure 1 and Figure 2). Together, this reaction sequence provides a complete oxidative degradation of glucose to 6 CO_2_ in 15 enzymatic steps, not much different from the canonic NADPH-producing pathway, the pentose phosphate pathway, which requires 12 steps for the same task (hexokinase plus seven core steps plus four steps of gluconeogenesis to recycle glyceraldehyde-3-phosphate and fructose-6-phosphate to glucose-6-phosphate). In this simplifying calculation, it is ignored that some enzymes are run through by several substrates, but this phenomenon occurs in both pathways almost equally. For comparison, the canonic coupling of glycolysis, pyruvate dehydrogenase, and the citric acid cycle achieves the same task in 19 steps (10 steps in glycolysis plus pyruvate dehydrogenase (counted as one step) plus 8 steps in the citric acid cycle).

A major difference in the three pathways is the fate of the 24 electrons gained from the complete oxidation of one molecule of glucose. As sketched in Scheme 2, the pentose phosphate pathway delivers 12 NADPH, the canonic pathway via the citric acid cycle delivers 10 NADH and 2 FADH_2_, and the complete serine–folate shunt will by default deliver 4 NADH and 8 NADPH. In other words, the latter may be viewed as a compromise between the two former pathways, as directly visible from Figure 1. Based on the widespread concomitant induction of fatty acid biosynthesis and β-oxidation in cells and animals after complex I inhibition, we recently proposed a model according to which NADPH is completely catabolically oxidized (by fatty acid cycling) to emergency fuel the respiratory chain (in the NADPH-FADH_2_ axis) [24]. Supposing a similar oxidation of the folate cycling-derived NADPH, a metabolic pathway emerges that might not be as energetically inefficient as the NADPH-FADH_2_ axis, but also not as entirely NAD-independent as the NADPH-FADH_2_ axis. It is of note that the NADPH-FADH_2_ axis is formally completely independent of oxidized NAD, which may indeed be severely reduced under conditions of complete complex I inhibition. A partial, but statistically significant loss of NAD and nicotinamide riboside has been demonstrated for the MetR rat liver [23], while in both cell culture models, marked increases in reduced NAD(P)H (generally involving a lower NAD/NADH ratio) have been noted [6,41]. The serine–phosphate shunt, however, spares approximately 60% of the limiting NAD by using NADP for electron acceptance. In support of our tentative coupling of the serine–folate shunt with fatty acid cycling is the finding of serine degradation-coupled fatty acid synthesis in the mouse liver [59].

Notably, in a typical in vivo situation as encountered in methionine restriction (i.e., hepatic complex I inhibition, but unaffected adipose tissue), the high metabolic cost of fatty acid cycling accrues exclusively in the adipose tissue, whereas the benefit of FADH_2_ oxidation accrues exclusively in the complex I-inhibited liver, enabling the latter to maintain its mitochondrial membrane potential [24]. Hence, energetically inefficient metabolic pathways may in fact provide a benefit to some cells and organs at the (potentially bearable) cost of other cells. Uncoupling respiration through UCP proteins is a prototypic example of such a negative ATP balance but is still associated with evolutionary and medical value, for instance, by preventing inner mitochondrial membrane hyperpolarization and the resulting excessive radical leak [60]. A similar argument has been put forward for “futile” metabolic cycles [61]. After all, metabolic inefficiency has been amply evidenced to occur after complex I inhibition [25,26,28].

An entirely unrelated, non-bioenergetic benefit of the proposed serine–folate shunt may be related to the avoidance of unduly increased intracellular glucose concentrations, which mediate many of the adverse effects of increased plasma glucose concentrations in diabetes [62]. Other potential strategies of “glucose detoxification” have been described before, including the enhancement of mitochondrial respiration [63] and the activation of glycerol-3-phosphate phosphatase [64].

Regarding any alternative beneficiaries of the induction of serinogenesis and folate metabolism, it appears that none of them is as universally applicable in the three paradigms analyzed in this study as is NADPH production for catabolic purposes. In particular, the current NADPH production does not seem to be related to oxidative stress repair, as only in the MPP-treated cells, was such stress evident. In the other two models, antioxidant enzymes were either unchanged (especially mitochondrial enzymes such as GPX4, SOD2, and TXN2) or downregulated (certain cytosolic enzymes such as GPX1, CAT, and TXN) (Table 2). These findings are line with the literature, which has reported modestly increased ROS production, but unchanged levels of lipid peroxidation and an unchanged thiol redox status in the patient fibroblasts examined here [41]. Similarly, in MetR animals, a substantial lowering of reactive oxygen species production has been reported [38,39,55], which might relate to the fact that these animals lived longer and did not show any broad induction of antioxidant enzymes (except GSR and TXNRD1) (Table 2) despite severe glutathione depletion and oxidation [23]. The folate-dependent pathway towards glutathione (via the methionine cycle and transsulfuration) was likewise unaltered in these animals (compare MTHFR, MTR, MAT2A, and CBS expression), and it was in fact severely downregulated in the apparently more stringent MPP-treated cell model. This makes a generalized demand for S-adenosylmethionine, methionine, cysteine, glutathione, or taurine an unlikely trigger of serinogenesis and folate cycling, despite numerous connections that could be envisaged. Serine is also not considered to be an antioxidant amino acid by itself [65].

A heightened demand for nucleotide biosynthesis requiring 10-formyl-THF is arguably the most prominent alternative to the here proposed interpretation of induced serinogenesis and folate metabolism, since the two 10-formyl-THF-dependent enzymes of purine biosynthesis (GART and ATIC) as well as the committed step enzyme PPAT and the salvage enzyme HPRT1 were variably induced in MPP-treated cells and MetR rats (Table 2). On the other hand, no such induction was seen in the patient fibroblast model, in which key enzymes of DNA synthesis were rather downregulated. Moreover, dihydrofolate reductase (DHFR) was likewise downregulated in both MPP-treated cells and MetR rats, which indicates that there was no heightened demand for polymeric DNA synthesis. Correspondingly, in both models, the enzymes of the oxidative, first part of the pentose phosphate pathway (to yield the dual-function intermediate ribulose-5-phosphate (R5P)) were less pronouncedly induced than the enzymes of the second part, which recycles excess R5P for further oxidation [24]. This finding is a general indicator that NADPH production is required by the cell rather than pentoses for nucleotide synthesis. At present, a specific demand for free purines appears to explain the observed transcriptional patterns best. Clearly, the situation may turn out to be different in rapidly dividing tumor cells with their elevated demand of nucleotides, which also frequently induce serinogenesis and the mitochondrial folate-metabolizing enzymes SHMT2 and MTHFD2 [57]. Further analyses should shed light on this issue and define the causes behind enhanced purine synthesis following complex I inhibition.

## 4. Materials and Methods

### 4.1. Model 1: MPP

The transcriptomic profile of in vitro-differentiated human dopaminergic neuronal stem cells (LUHMES cells) after complex I inhibitory MPP treatment was generated as described [6] and has been deposited in the GEO repository (GSE229460). Data were from 3 vs. 3 cell culture replicates. In brief, the cells had been cultivated on polyornithine/fibronectin-coated plastic dishes in DMEM/F12 (50:50) medium containing 1× N2 supplement and 0.04 mg/mL bFGF at 37 °C under a humidified atmosphere containing 5% CO_2_. After 4 days of differentiation with 1 mM cAMP, 1 mg/mL tetracycline and 2 ng/mL GDNF, the cells were treated with 10 µM of the complex I inhibitor [66] 1-methyl-4-phenylpyridinium iodide (MPP) or vehicle for 48 h [29,53]. Under the employed conditions of MPP treatment, these cells have been evidenced to exhibit an increased NADH/NAD ratio, increased glucose consumption and lactate production as well as increased ROS production, but unaltered ATP levels [6].

### 4.2. Model 2: MetR

Processed transcriptomic and metabolomic data from a cohort of male, methionine-restricted Fischer F344 rats have been published [23]. The original data were kindly provided by Carmen E. Perrone and Jay A. Zimmerman (Orentreich Foundation for the Advancement of Science, Inc., Cold Spring-on-Hudson, NY, USA). The currently analyzed data were from adult liver specimens of 6 vs. 6 animals. Individually caged Fischer F344 rats had been placed on a methionine-restricted diet for three months duration starting at an age of six weeks, as detailed [23]. Methionine restriction was achieved using chemically defined diets in which nutritional protein had been replaced by amino acid mixtures containing either 0.86% methionine (control diet) or 0.17% methionine (methionine-restricted diet). Food and water were provided ad libitum. These animals have been extensively characterized and shown to exhibit an antidiabetic, slim, hyperphagous, metabolically inefficient, but long-lived phenotype with numerous endocrinological and enzymological phenotypes [23,27,40].

### 4.3. Model 3: NDUFS2

RNA sequencing data from cultivated skin biopsy fibroblasts sampled from a patient (ID #5170) carrying a homozygous NDUFS2 mutation (R228Q) versus control patient fibroblasts (ID #4996) were obtained from the GEO repository (GSE65634) [42]. Data were from 2 vs. 2 cell culture replicates. Propositus cells and control cells had been cultivated in M199 medium supplemented with 10% fetal calf serum and penicillin/streptomycin. The cells were harvested at confluency after 72 h incubation in medium with 0.01% DMSO. Cells from this patient had 39% residual CI activity, approximately doubled ROS production, but unaltered levels of lipid peroxidation and an unaltered glutathione redox steady state [41]. An increase in NAD(P)H autofluorescence has also been described [41], which would be predictive of citric acid cycle inhibition [4].

Widespread compensatory induction of nuclear-encoded respiratory chain complex genes has been evidenced for MPP-treated LUHMES cells [6] and methionine-restricted rats [40], whereas patient fibroblasts harboring complex I mutations have behaved variably in this respect [42].

### 4.4. Technical and Statistical Parameters

Statistical significance thresholds were generally adopted from the original data sources. An identical threshold as in model 1 was applied to model 3 (i.e., Benjamini–Hochberg-adjusted *p* values < 10^−6^ without defining any minimum fold changes) because of the similar technical setup and experimental design (multiplex RNA sequencing data from cell culture replicates analyzed with the Bioconductor package DESeq2) [67]. Due to the etiologically different nature of the three datasets, statistical cross-comparisons between the models should be avoided; however, within each model, *p* values were coherent and can be used to evaluate the relative regulation of different genes. Quantitative cross-comparisons of the fold changes should be conducted with due care. A brief technical description of each experiment is provided in the following.

Model 1. Multiplex RNA sequencing was performed on an Illumina NextSeq 500 platform (Illumina, San Diego, CA, USA). Reads were processed with Illumina bcl2fastq and FastQC and stepwise filtered for quality, mismatches with the human reference genome GRCh38 and secondary alignments as described [6]. Of the raw 58,038 RNAs detected by sequencing, 10,924 were assigned to genes expressed at a level of more than 1 RPKM (read per kilobase of transcript per million mapped reads) using the Bioconductor package DESeq2 v.1.18.1 [67], which was also employed to calculate fold changes (FCs) and Benjamini–Hochberg-adjusted *p* values. Of the 10,924 final reference genes, 6034 were significantly modulated at the *p* < 10^−6^ level (n = 3).

Model 2. Transcriptomic analyses were conducted with manually synthesized cDNAs applied to Affymetrix Rat Exon ST 1.0 Arrays (containing approximately 10^6^ probes, including probes for 92,038 putative full-length mRNAs and 850,000 exon clusters) (Affymetrix, Santa Clara, CA, USA). The raw microarray data were normalized and filtered using GeneSpring GX v.11 as described [23], annotated using Expression Console from Affymetrix and further evaluated by ANOVA with a Benjamini–Hochberg false discovery rate correction to identify differentially expressed genes. Of the 12,826 genes passing the filtering steps, an adopted significance threshold of *p* < 0.05 (n = 6) identified 2170 regulated genes. Due to the technical discrepancy between the current microarray experiment and the multiplex RNA sequencing approaches of the other two models, the available TaqMan quantitative PCR data published in the same study [23] were also evaluated. Such data could be recovered for eight key genes and represent n = 7–8 animals [23].

Model 3. Multiplex RNA sequencing was performed on an Illumina HiSeq2000 platform. Reads were filtered for adaptors, sequence length, and mismatches to the reference genome as described [42]. Of the 10,270,698 short reads passing the filtering, 19,426 reference gene transcripts were identified that were detected in at least one sample, of which 14,592 were expressed at a baseMean level (as per DESeq2 v.1.40.2) [67] of more than five, which entered the statistical analysis. Of the 14,592 final reference genes, 1460 were significantly regulated at the *p* < 10^−6^ level (n = 2) (Benjamini–Hochberg-adjusted *p* values). Fold changes (FCs) and *p* values were also calculated with DESeq2 as in model 1.

## 5. Conclusions

Quantitative induction of serinogenesis is a recurring stress response to various forms of respiratory chain inhibition. The ultimate meaning of this rather isolated induction of an amino acid biosynthetic pathway in the wake of mitochondrial impairment has remained unsettled, since multiple catabolic and anabolic purposes, both folate-dependent and folate-independent, have appeared plausible. The current transcriptomic comparison of three divergent models of selective complex I inhibition indicates that the universal purpose of quantitative serinogenesis is the catabolic degradation of excess glucose (“glucose detoxification”), coupled with an emergency fueling of the respiratory chain downstream of the inhibited complex I (via the “serine–folate shunt”). Oxidative stress repair and the provision of methionine, cysteine, glutathione, and taurine as well as directly serine-dependent anabolic pathways do not widely accompany serinogenesis and thus fall short of providing a general explanation. The serine–folate shunt represents an alternative catabolic avenue of complete glucose oxidation to CO_2_ beyond the canonic pathways (i) glycolysis/citric acid cycle, and (ii) pentose phosphate pathway.

## Figures and Tables

**Table 1:** Expression of genes coding for proteins of the serinogenesis pathway, folate cycling, and associated proteins.

		MPP Treatment		Dietary Methionine Restriction (MetR)		Hereditary Complex I Deficiency (NDUFS2)	
	Gene	FC	*p* Value	FC *FC* (*PCR*)	*p* Value *p Value*	FC	*p* Value
**Serine metabolism**	*PHGDH*	**7.42**	1 × 10^−112^	**1.76** ***9.50***	1 × 10^−5^ *<0.001*	**9.25**	1 × 10^−104^
	*PSAT1*	**2.94**	3 × 10^−39^	**2.64**	1 × 10^−5^	**12.64**	1 × 10^−132^
	*PSPH*	0.69	1 × 10^−5^	**1.39**	3 × 10^−3^	1.75	1 × 10^−3^
	*SFXN1*	**1.67**	3 × 10^−15^	0.97	4 × 10^−1^	1.23	9 × 10^−2^
	*SLC25A38*	**2.56**	1 × 10^−24^	—	—	1.11	5 × 10^−1^
**Folate cycling**	*SHMT1*	0.52	6 × 10^−6^	1.05	2 × 10^−1^	1.15	5 × 10^−1^
	*SHMT2*	**4.56**	6 × 10^−93^	1.07	6 × 10^−2^	**1.94**	2 × 10^−13^
	*MTHFD1*	**1.76**	1 × 10^−8^	**1.38** ***1.89***	4 × 10^−2^ *<0.001*	0.77	6 × 10^−3^
	*MTHFD1L*	**8.85**	1 × 10^−98^	**1.08**	3 × 10^−2^	**2.01**	3 × 10^−18^
	*MTHFD2*	**9.06**	4 × 10^−124^	**4.54** ***11.68***	1 × 10^−6^ *<0.001*	**3.48**	2 × 10^−30^
	*MTHFD2L*	[1.19]	2 × 10^−1^	—	—	0.46	1 × 10^−1^
	*ALDH1L1*	[4.13]	1 × 10^−4^	—	—	—	—
	*ALDH1L2*	1.40	5 × 10^−5^	—	—	**1.80**	1 × 10^−14^
**Glycine cleavage** **system**	*GLDC*	1.14	1 × 10^−1^	**1.63** ***2.29***	3 × 10^−3^ *<0.001*	—	—
	*AMT*	[41.64]	2 × 10^−4^	—	—	1.20	4 × 10^−1^
	*GCSH*	[1.05]	9 × 10^−1^	1.01	9 × 10^−1^	1.24	3 × 10^−1^
	*DLD*	**0.52**	5 × 10^−14^	**1.08**	3 × 10^−3^	1.04	7 × 10^−1^
**NADPH** **export**	*IDH1*	**1.40**	6 × 10^−7^	1.00	9 × 10^−1^	0.99	9 × 10^−1^
	*IDH2*	**1.93**	9 × 10^−17^	1.33	3 × 10^−2^	0.89	3 × 10^−1^

Significant fold changes (FCs) are marked in bold type. Significance thresholds were assigned as follows: MPP treatment, *p* = 1 × 10^−6^; dietary methionine restriction, *p* = 5 × 10^−2^; hereditary complex I deficiency, *p* = 1 × 10^−6^. Italics label manually generated TaqMan PCR results, as opposed to DNA microarray and RNA sequencing data. Dashes indicate that the respective genes could not be recovered from the original dataset, generally due to low expression levels. Parentheses were used in the MPP dataset when baseline gene expression was below the quality threshold of 1 RPKM; these data should be interpreted cautiously. Gene abbreviations in the order of appearance are as follows: PHGDH, phosphoglycerate dehydrogenase; PSAT1, phosphoserine aminotransferase 1; PSPH, phosphoserine phosphatase; SFXN1, sideroflexin 1; SLC25A38, solute carrier family 25 member 38; SHMT1, serine hydroxymethyltransferase 1; SHMT2, serine hydroxymethyltransferase 2; MTHFD1, methylenetetrahydrofolate dehydrogenase, cyclohydrolase, and formyltetrahydrofolate synthetase 1; MTHFD1L, methylenetetrahydrofolate dehydrogenase (NADP-dependent) 1 like; MTHFD2, methylenetetrahydrofolate dehydrogenase (NADP-dependent) 2, methenyltetrahydrofolate cyclohydrolase; MTHFD2L, methylenetetrahydrofolate dehydrogenase (NADP-dependent) 2 like; ALDH1L1, aldehyde dehydrogenase 1 family member L1; ALDH1L2, aldehyde dehydrogenase 1 family member L2; GLDC, glycine decarboxylase; AMT, aminomethyltransferase; GCSH, glycine cleavage system protein H; DLD, dihydrolipoamide dehydrogenase; IDH1, isocitrate dehydrogenase (NADP) 1; IDH2, isocitrate dehydrogenase (NADP) 2.

**Table 2:** Expression of genes coding for proteins that require serine, C_1_-loaded folate, or NADPH as substrates, as well as associated genes.

		MPP Treatment		Dietary Methionine Restriction (MetR)		Hereditary Complex I Deficiency (NDUFS2)	
	Gene	FC	*p* Value	FC *FC (PCR)*	*p* Value *p Value*	FC	*p* Value
Serine usage *	*SRR* *	0.75	2 × 10^−3^	1.00	1 × 10^−0^	0.87	3 × 10^−1^
	*SPTLC1* *	1.03	8 × 10^−1^	0.99	8 × 10^−1^	1.14	2 × 10^−1^
	*SPTLC2* *	1.08	3 × 10^−1^	—	—	1.34	2 × 10^−3^
	*CBS* *	[1.86]	3 × 10^−5^	0.87 ***1.22***	7 × 10^−2^ *0.03*	1.23	9 × 10^−2^
Folate usage * and related genes	*MTFMT* *	**0.36**	4 × 10^−17^	1.07	5 × 10^−1^	1.01	9 × 10^−1^
	*TYMS* *	0.79	1 × 10^−1^	1.16	8 × 10^−2^	**0.55**	1 × 10^−7^
	DHFR*	**0.23**	2 × 10^−27^	**0.41**	5 × 10^−2^	0.68	1 × 10^−3^
	*CAD*	1.00	1 × 10^−1^	**1.19**	2 × 10^−6^	1.16	1 × 10^−1^
	*GART* *	1.04	5 × 10^−1^	**1.25**	1 × 10^−2^	1.43	2 × 10^−4^
	*ATIC* *	**2.25**	2 × 10^−24^	**1.44**	2 × 10^−3^	1.15	1 × 10^−1^
	*PPAT*	1.24	3 × 10^−2^	**1.57** ***2.64***	8 × 10^−4^ *<0.001*	0.85	5 × 10^−1^
	*APRT*	1.03	2 × 10^−2^	1.08	1 × 10^−1^	0.95	7 × 10^−1^
	*HPRT1*	**2.15**	9 × 10^−12^	**1.33**	3 × 10^−3^	1.09	6 × 10^−1^
	*RRM1*	**1.52**	2 × 10^−7^	1.07	6 × 10^−2^	0.77	6 × 10^−3^
	*RRM2*	0.90	6 × 10^−1^	—	—	**0.53**	8 × 10^−9^
	*MTHFR* *	**0.18**	1 × 10^−89^	1.02 *1.17*	6 × 10^−1^ *0.49*	0.95	7 × 10^−1^
	MTR*	0.62	4 × 10^−6^	1.16 ***1.75***	1 × 10^−1^ *<0.001*	0.99	1 × 10^−0^
	*MAT2A*	**0.12**	4 × 10^−149^	0.92	5 × 10^−1^	0.90	2 × 10^−1^
**NADPH** usage * and related genes	*GSR* *	**3.05**	1 × 10^−43^	**1.69**	4 × 10^−4^	1.40	2 × 10^−3^
	*GPX1*	**2.80**	7 × 10^−30^	**0.76**	7 × 10^−4^	**0.48**	1 × 10^−18^
	*GPX4*	**2.77**	3 × 10^−40^	1.04	6 × 10^−1^	1.23	1 × 10^−2^
	*SOD1*	**2.07**	5 × 10^−16^	0.95	8 × 10^−2^	0.99	9 × 10^−1^
	*SOD2*	1.34	3 × 10^−4^	1.03	8 × 10^−1^	1.17	5 × 10^−2^
	*CAT*	**3.09**	3 × 10^−39^	**0.89**	3 × 10^−3^	0.69	2 × 10^−5^
	*TXNRD1* *	1.35	6 × 10^−6^	**1.43**	7 × 10^−5^	**1.95**	5 × 10^−24^
	*TXNRD2* *	[0.66]	7 × 10^−3^	—	—	0.76	1 × 10^−1^
	*TXN*	**1.62**	2 × 10^−9^	0.92	2 × 10^−1^	**0.63**	5 × 10^−9^
	*TXN2*	**2.20**	5 × 10^−21^	1.02	9 × 10^−1^	1.09	4 × 10^−1^
	*MSRA* *	**1.84**	9 × 10^−9^	—	—	0.95	8 × 10^−1^
	*MSRB1* *	**0.26**	2 × 10^−53^	—	—	1.22	2 × 10^−1^
	*MSRB2* *	1.31	2 × 10^−3^	0.87	2 × 10^−2^	1.43	5 × 10^−3^

Asterisks label gene products that directly consume the listed factors serine, folate, or NADPH, respectively. Significant fold changes (FCs) are marked in bold type. Significance thresholds were adopted as before: MPP treatment, *p* = 1 × 10^−6^; dietary methionine restriction, *p* = 5 × 10^−2^; hereditary complex I deficiency, *p* = 1 × 10^−6^. Italics label TaqMan PCR results, dashes indicate a lack of corresponding data, and parentheses denote a low baseline gene expression (<1 RPKM), as detailed in Table 1. Gene abbreviations in the order of appearance are as follows: SRR, serine racemase; SPTLC1, serine palmitoyltransferase long chain base subunit 1; SPTLC2, serine palmitoyltransferase long chain base subunit 2; CBS, cystathionine beta-synthase; MTFMT, mitochondrial methionyl-tRNA formyltransferase; TYMS, thymidylate synthetase; DHFR, dihydrofolate reductase; CAD, carbamoyl-phosphate synthetase 2, aspartate transcarbamylase, and dihydroorotase; GART, phosphoribosylglycinamide formyltransferase, phosphoribosylglycinamide synthetase, phosphoribosylaminoimidazole synthetase; ATIC, 5-aminoimidazole-4-carboxamide ribonucleotide formyltransferase/IMP cyclohydrolase; PPAT, phosphoribosyl pyrophosphate amidotransferase; APRT, adenine phosphoribosyltransferase; HPRT1, hypoxanthine phosphoribosyltransferase 1; RRM1, ribonucleotide reductase catalytic subunit M1; RRM2, ribonucleotide reductase regulatory subunit M2; MTHFR, methylenetetrahydrofolate reductase; MTR, 5-methyltetrahydrofolate-homocysteine methyltransferase; MAT2A, methionine adenosyltransferase 2A; GSR, glutathione-disulfide reductase; GPX1, glutathione peroxidase 1; GPX4, glutathione peroxidase 4; SOD1, superoxide dismutase 1; SOD2, superoxide dismutase 2; CAT, catalase; TXNRD1, thioredoxin reductase 1; TXNRD2, thioredoxin reductase 2; TXN, thioredoxin; TXN2, thioredoxin 2; MSRA, methionine sulfoxide reductase A; MSRB1, methionine sulfoxide reductase B1.

## Data Availability

The original contributions presented in this study are included in the article. Further inquiries can be directed to the corresponding author.
